# PREACT-digital: study protocol for a longitudinal, observational multicentre study on digital phenotypes of non-response to cognitive behavioural therapy for internalising disorders

**DOI:** 10.1136/bmjopen-2025-102392

**Published:** 2025-07-10

**Authors:** Leona Hammelrath, Annette Brose, Manuel Heinrich, Pavle Zagorscak, Sebastian Burchert, Till Langhammer, Christine Knaevelsrud

**Affiliations:** 1 Department of Clinical Psychological Intervention, Freie Universität Berlin, Berlin, Germany; 2 Freie Universitat Berlin, Berlin, Germany; 3 Clinical-Psychological Intervention, Freie Universität Berlin Fachbereich Erziehungswissenschaft und Psychologie, Berlin, Germany; 4 Psychology, Humboldt-Universitat zu Berlin, Berlin, Germany; 5 Department of Clinical Psychological Intervention and Psychotherapy, Freie Universität Berlin, Berlin, Germany

**Keywords:** Machine Learning, Digital Technology, Anxiety disorders, Depression & mood disorders

## Abstract

**ABSTRACT:**

**Introduction:**

Cognitive behavioural therapy (CBT) serves as a first-line treatment for internalising disorders (ID), encompassing depressive, anxiety or obsessive-compulsive disorders. Nonetheless, a substantial proportion of patients do not experience sufficient symptom relief. Recent advances in wearable technology and smartphone integration enable new, ecologically valid approaches to capture dynamic processes in real time. By combining ecological momentary assessment (EMA) with passive sensing of behavioural and physiological information, this project seeks to track daily fluctuations in symptom-associated constructs like affect, emotion regulation (ER) and physical activity. Our central goal is to determine whether dynamic, multimodal markers derived from EMA and passive sensing can predict treatment non-response and illuminate key factors that drive or hinder therapeutic change.

**Methods and analysis:**

PREACT-digital is a subproject of the Research Unit FOR 5187 (PREACT), a large multicentre observational study in four outpatient clinics. PREACT channels state-of-the-art machine learning techniques identify predictors of non-response to CBT in ID. The study is currently running and will end in June 2026. Patients seeking CBT at one of four participating outpatient clinics are invited to join PREACT-digital. They can take part in (1) a short version with a 14-day EMA and passive sensing phase prior to therapy, or (2) a long version in which the short version’s assessments are extended throughout the therapy. It is estimated that 468 patients take part in PREACT-digital, of which 350 opt for the long version of the study. Participants are provided with a smartwatch and a customised study app. We collect passive data on heart rate, physical activity, sleep and location patterns. EMA assessments cover affect, ER strategies, context and therapeutic agency. Primary outcomes on (non)-response are assessed after 20 therapy sessions and therapy end. We employ predictive and exploratory analyses. Predictive analyses focus on classification of non-response using basic algorithms (ie, logistic regression and gradient boosting) for straightforward interpretability and advanced methods (LSTM, DSEM) to capture complex temporal and hierarchical patterns. Exploratory analyses investigate mechanistic links, examine the interplay of variables over time and analyse change trajectories. Study findings will inform more personalised and ecologically valid approaches to CBT for ID.

**Ethics and dissemination:**

The study has received ethical approval from the Institutional Ethics Committee of the Department of Psychology at Humboldt Universität zu Berlin (Approval No. 2021–01) and the Ethics Committee of Charité-Universitätsmedizin Berlin (Approval No. EA1/186/22). Written informed consent will be obtained from all participants prior to enrolment. Results will be disseminated through peer-reviewed journals and presentations at national and international conferences.

**Trial registration number:**

DRKS00030915; OSF PREACT: http://osf.io/bcgax; OSF PREACT-digital: https://osf.io/253nb.

**Strengths and limitations of this study**

- Large sample of clinical patients starting cognitive behavioural therapy, which is rare in digital phenotyping studies.
- Longitudinal assessment combining passive sensing and ecological momentary assessment, capturing a dynamic, multidimensional view of participants’ behaviours, emotions and physiological states, and enabling analysis of within-person trajectories.
- State-of-the-art, consumer-grade wearables (Withings Scanwatch) for all participants, ensuring high-quality, standardised data collection across participants and ensuring the privacy of participants by not disclosing them as study participants.
- There is no healthy control group, which limits the ability to distinguish clinical from non-clinical patterns in digital phenotyping data.
- Participant burden due to extensive assessments might decrease adherence and influence therapy outcomes.

## Introduction

 The following study protocol describes a subproject from the Research Unit FOR 5187 ‘Towards Precision Psychotherapy for Non-Respondent Patients: From Signatures to Predictions to Clinical Utility (PREACT)’ that was formed as a collaboration across four universities and outpatient clinics in Berlin to identify predictors of non-response to cognitive behavioural therapy (CBT) in Internalising Disorders (ID). The diagnostic and experimental focus of PREACT lies on emotion regulation (ER) as a putative key mechanism of CBT and response to CBT.

Due to the comprehensive design of the Research Unit, the general study protocol provides an overview across all subprojects.^1^ The present protocol entails a more granular description of methods and objectives relevant to this subproject. This level of detail is of utter importance to ensure that reporting recommendations are met^2^ and researchers can assess the comparability with other digital phenotyping studies. Associated materials and descriptions can be found in our Open Science Framework (OSF) directory (https://osf.io/253nb) and on Github (https://github.com/leona-ha/preact_digital).

ID encompass a broad range of psychopathology, including symptoms of depressive disorder, obsessive-compulsive disorder or generalised anxiety disorder.^3^ They represent the most common mental health conditions worldwide.^4^ CBT is considered the first-line treatment for ID but was shown to produce insufficient response rates.^5^ In addition, urgently needed breakthroughs in psychotherapy research remain elusive: studies adapting or enhancing CBT or comparing it to other forms of psychotherapy for ID, like behavioural activation therapy or interpersonal therapy, often do not find significant differences in outcomes.^6^

A great hope lies within the field of machine learning, which has found its way into psychotherapy research within recent years.^7^ Here, a prominent approach is to predict outcomes before the treatment started using a large number of different variables from various sources.^8 9^ The idea is to change or adapt a treatment if the risk of non-response is high and thus avoid frustrating experiences for patients as well as unnecessary societal and economical burdens. Thus far, however, results are unsatisfactory: depending on the features of input, predictive accuracies often only slightly exceed chance level, like 62% for neuroimaging markers^10^ and/or fail to generalise to external samples.^11^

Most existing prediction studies relied on cross-sectional data (ie, pre-treatment self-reports). However, these momentary snapshots are incapable of depicting the intraindividual symptom heterogeneity therein.^12 13^ Beyond that, the applicability to outpatient care—as an ultimate goal of precision psychotherapy—is neglected when relying solely on time and cost-intensive forms of phenotyping like clinical interviews or neurophysiological markers.^14^

Luckily, recent advances in wearable devices and smartphone technology and the broad integration of smartphones into people’s everyday life are paving the way for more accessible, ecologically valid data collection methods. In particular, these tools offer a scalable alternative that can reduce reliance on labour-intensive procedures. Ecological momentary assessment (EMA) involves short, repeated self-reports on the smartphone. Passive sensing refers to the continuous collection of biobehavioural data (ie, physical activity and heart rate) using smartphone or wearable sensors. The combination of active (ie, EMA-based) and passive (ie, wearable-based) assessments is also referred to as ‘Digital phenotyping’.^15^ Digital phenotyping offers the opportunity to better understand individual factors and mechanisms leading to (non-) response by (a) opening the black box of between-session processes and gathering naturalistic information from everyday settings, (b) getting long-term subjective and objective impressions of symptom dynamics in (c) real time and without recall biases inherent to cross-sectional assessments.

First, systematic reviews on digital phenotyping in different ID concluded that, although in its infancy, it can identify behavioural patterns associated with ID.^16 17^ These include, for example, increased time at home in depression^18 19^ or a positive association between heart rate variability (HRV) and anxiety.^20^ At the same time, only a few studies have applied digital phenotyping (DP) in patients undergoing psychotherapy for ID to depict (absent) changes in symptoms and/or predict treatment response. De Angel *et al*^21^ collected DP data from 66 patients starting psychological treatment for depression. They only published results on the feasibility of data collection, showing that data availability varied strongly depending on data source (ie, smartphone vs wearable) and treatment stage (ie, pretreatment vs in-treatment vs posttreatment). Müller-Bardoff *et al*^22^ conducted a randomised-controlled trial (RCT) to find sensor-based and EMA-based predictors of response to CBT in 150 patients suffering from anxiety disorders. To date, they did not publish results related to DP-based outcome prediction. In psychiatric settings, Zou *et al*^23^ collected passive sensor data (ie, phone usage, app usage and sleep) from 245 patients with major depressive disorder to predict response to psychopharmacological outpatient treatment. They were able to achieve sufficient predictive accuracy around 10 weeks before treatment ended.

In summary, there remains a need for studies investigating digital phenotyping as both predictors and markers of treatment response in ID. Our subproject, PREACT-digital, addresses this gap. We implement EMA and passive sensing via state-of-the-art wearable devices to continuously monitor patients receiving CBT for ID in their daily lives. Building on the PREACT consortium’s overarching focus on ER, we incorporate ER-related constructs such as affect and social/situational context into our EMA measures. Ultimately, our goal is to identify ecologically valid, readily implementable markers of treatment response—or the absence thereof—in CBT for ID.

Our research objectives encompass predictive and exploratory hypotheses. First, in-line with PREACT, we aim to find out if we can predict non-response (NR) at T20 (ie, after 20 therapy sessions) and TPost (ie, after therapy end or 365 days if therapy lasts longer) with sufficient predictive accuracy (i.e. >75%) using a combination of EMA and passive sensing features collected during the first, 14-day assessment phase (T0). We expect that EMA-based features can better capture ER-related dynamics and are thus of greatest importance, but passive features will provide significant incremental value. We further hypothesise that EMA and sensing features both have an incremental predictive value beyond more elaborate (neuro)physiological markers and thus represent an ecologically valid substitute for implementation. Second, we aim to find out if symptom changes during CBT can be modelled using EMA and passive sensing, and how they relate to cross-sectional self-report and physiological information. We hypothesise that data actively and passively generated by personal electronic devices such as smartphones and wearables can be linked to neuroimaging-based markers of ER assessed in other subprojects of the research group.

## Method and analysis

### Sample selection

Participants within the PREACT study are informed about PREACT-digital via flyers and information brochures within each of the participating outpatient clinics. Interested patients are invited to an ‘onboarding’ meeting to review inclusion and exclusion criteria and provide written informed consent. The inclusion criteria of the PREACT study are as follows: (1) age of 18 years or older, (2) a primary diagnosis based on the DSM-5 criteria for either social anxiety disorder, panic disorder, agoraphobia, generalised anxiety disorder, obsessive-compulsive disorder, posttraumatic stress disorder or unipolar depressive disorder (major depression or dysthymia), (3) an indication for outpatient CBT treatment, (4) a minimum treatment plan of 12 sessions, (5) a symptom severity indicated by a General Severity Index of the Brief Symptom Inventory (BSI-GSI) greater than 0.56 (19, 20), (6) a Clinical Global Impression – Severity Scale (GSI-S) score of 3 or higher, indicating at least mild illness, and (7) owning an appropriate smartphone (ie, android version >9.0 or i-Phone version >15.0).

Exclusion criteria encompass contraindications for outpatient treatment, including: (1) a current secondary diagnosis of moderate to severe substance use disorder (including regular use of benzodiazepines), (2) current psychosis, (3) current bipolar disorder, (4) more than moderate suicidality, (5) medical conditions that contraindicate CBT according to the medical consultation report (ie, severe cognitive impairment or dementia). Patients who require inpatient treatment during the course of the outpatient treatment will be excluded.

### Study procedures

**Figure 1:**
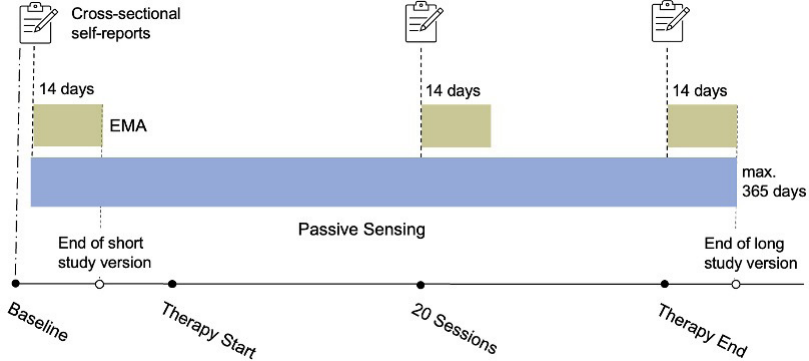
Study procedures. EMA, ecological momentary assessment.

The PREACT-digital study is currently running and will end in June 2026. Interested patients can choose if they want to take part in PREACT-digital (ie, therapy-accompanying digital phenotyping). Individuals who decide to participate in the PREACT-digital can choose between two study versions. Option 1 (‘the short version‘) consists of a 14-day EMA assessment phase paralleled by passive data collection prior to therapy start (T0) only. Option 2 (‘the long version’) consists of one additional EMA phase after 20 therapy sessions (T20) and one after the end of therapy (if the duration of therapy exceeds 1 year, data are collected 365 days after therapy start; TPost). Passive data are collected in parallel throughout the entire time. Participants enrolled in the short version have the option to switch to the long version after they have completed the first assessment phase. The design of our subproject is depicted in figure 1.

Once enrolled in PREACT-digital, participating patients go through an onboarding process with trained research assistants. Together, they instal the required apps and connect the smartwatch. For passive data collection in PREACT-digital, patients receive a state-of-the-art smartwatch (Withings Scanwatch Light), the associated Withings app and a customised study app developed by a German tech-startup, called TIKI-app. The TIKI app (1) serves as an interface to the Withings–API, allowing access to the smartwatch data (2) sends out the EMA questionnaires and (3) collects GPS data.

Participants receive information and materials for study participation and provide additional electronic informed consent via the study app. During the first measurement burst, patients receive an onboarding call from the study team, where they get the option to ask questions and report technical problems. In addition, the acceptability and feasibility of EMA and passive sensing are assessed as part of the onboarding call (see interview guideline on https://osf.io/253nb). Information gathered during the onboarding call is entered into a respective REDCap sheet. Between the active assessment phases, patients receive regular update mails with information about their last EMA completion rate and subsequent study procedures.

To reduce interruptions in data transmission leading to high rates of missing data, we implement weekly data monitoring. We run a script to identify participants who have not provided data for more than 7 days. If GPS and/or passive sensing data are absent for more than 7 days, the respective participants receive an e-mail containing instructions on how to restore data transmission. If data transmission is not restored in the following week, participants are contacted by phone. Reasons for interruptions (ie, app settings, participant forgetting to charge phone) are noted, when available. In addition, all active patients receive a notification on Sunday evenings asking them to open the TIKI-App and check settings to avoid automatic suppression of data collection.

Participants can decide to stop participation in PREACT-digital at any time point. In case of dropout, participants are contacted via mail to assess reasons for dropout.

### EMA assessment schedule

During the 14 days of active assessments, patients receive notifications (‘beeps’) in the TIKI-App on their smartphones eight times a day at quasirandom intervals of 90±30 min. Depending on their individual sleep—wake rhythm, participants can choose to receive beeps between 7.30 and 21.30 or 09.30 and 22.30. The respective questionnaires expire after 30 min. Each EMA assessment contains 30–35 items in total, depending on assessment phase and timing. The order of items was pseudorandomised.

We employ graded reimbursement, as it was shown to increase compliance: participants received at least 20€ per assessment phase, 35€ if they completed at least 90 beeps and 50€ if they completed more than 100 beeps. Thus, participants receive up to 150€ for the EMA assessments, which are paid out as a gift voucher. Moreover, individuals participating in the long version of the study are allowed to keep the smartwatch.

### EMA measures

Table 1 contains an overview of all measures. The whole set of items, including response options and assessment schedules, is provided online. Items are presented in German, but example items were translated to English here to facilitate comprehension for non-German-speaking readers. The following constructs are assessed:

- *Affect*: Participants were asked about their current affective state. The 17 items contain the PANAS-X (21) subscales, with additional single items on loneliness, fatigue and shyness. At each prompt, they were asked to rate on a scale from 1 to 7 how much they currently felt the respective affect.
- *Emotion Regulation*: At each beep, participants were asked to rate the intensity and controllability of their most negative thought since the last beep. Then we assessed the use of different ER strategies since the last beep using a German translation of the Regulation of Emotion Systems (RESS–EMA) scale,^24^ with six items covering reappraisal, rumination, suppression, distraction and relaxation. We implemented an additional item on acceptance.
- *Situational Context:* Self-constructed. To assess the situational context, we developed a custom measurement. Participants were asked to specify activities they had pursued in the preceding 2 hours from a given set of nine common activities. Participants were able to select multiple options simultaneously. The options were influenced by the DIAMONS scale and a comparable long-term digital phenotyping study.^25 26^ Here, we aimed to find a balance between sparsity of items and a high degree of situational coverage. Subsequently, they were asked to evaluate how much they enjoyed the respective activities.
- *Social Context:* Self-constructed. Participants were asked if they had social contacts since the last beep (online/ in person/ phone) and how agreeable the contact was.
- *Significant Events:* Self-constructed. Patients were asked to think about the most important event since the last beep and how pleasant they perceived it.
- *Physical fitness:* Self-constructed. Participants were asked how physically healthy they had felt today on the last beep of the day.
- *Therapeutic Agency*: We constructed four items based on the Therapeutic Agency Inventory (TAI)^27^ to assess Therapeutic Agency (TA) in everyday life. The original TAI contains three subscales, covering in-session activities, passivity towards the therapist and out-of-session activities. As we were interested in assessing TA in everyday life, our TAI–EMA items are based on the ‘out-of-session activities‘ subscales and cover cognitive and behavioural aspects of TA.
- *ECG Control:* During measurement bursts, patients were asked two times day to conduct a resting-state ECG on their Scanwatch. To control for potential confounders influencing the signal, we asked if they had consumed nicotine, caffeine or alcohol or had a heavy meal in the last 30 min.

**Table 1:** Overview of data to be collected

Domain	Construct	Description	Example feature	N items	Timing, Sampling
EMA	Positive and Negative Affect	PANAS-X+item on loneliness	How anxious do you feel right now?	17	Baseline, T20, TPost, all beeps
	Emotion Regulation	RESS–EMA scale+item on acceptance	In reaction to the negative feeling I…tried to breathe deeply	8	Baseline, T20, TPost, all beeps
	Situational Context	Self-constructed	How did you spend the time since the last beep?	1	Baseline, T20, TPost, all beeps
	Social Context	Self-constructed	Did you have social contact since the last beep?	3	Baseline, T20, TPost, all beeps
	Significant Events	Self-constructed	How did you perceive the most significant moment since the last beep?	1	Baseline, T20, TPost, all beeps
	Physical Health	Self-constructed	How physically healthy did you feel today?	1	Baseline, T20, TPost, last beep
	Therapeutic Agency	Constructed based on TAI	Today I have… implemented ideas or tasks from therapy	4	T20, TPost, last beep
	ECG Control	Self-constructed	Did you consume coffee in the last 30 min?	1	Baseline, T20, TPost, third and fifth beep
Sensing	GPS	Raw; TIKI-App	Time spent at home		Continuous; event-based
	Steps	Interval sum; Scanwatch	N steps		Continuous; event-based
	Activity	Scanwatch	Time spent cycling, calories burned		Continuous; event-based
	Heart rate	PPG; Interval mean; Scanwatch	Average heart rate		Continuous; regular 30 s samples
	ECG	Resting-State ECG. Scanwatch	Heart Rate Variability		Baseline, T20, TPost; 2 x daily; 30 s
	Sleep	Aggregate scores; Scanwatch	Time in Bed		Continuous

EMA, ecological momentary assessment; PANAS, positive and negative affect; PPG, photoplethysmography; T20, after 20 therapy sessions; TPost, after therapy end or 365 days if therapy lasts longer.

### Passive sensing data

We use a Withings Scanwatch to collect passive data on sleep, physical activity and heart rate (see table 1 for an overview). The following sensor data are available: for sleep, we have SleepDeepBinary, SleepLightBinary, SleepREMBinary, SleepStateBinary, SleepBinary, SleepInBedBinary and SleepAwakeBinary. They indicate if a user was, for a given time period, in deep sleep, light sleep, REM sleep, any sleep state, asleep overall, in bed (whether asleep or awake) or awake. For physical activity, we have ActivityType, ActiveBinary, RunBinary, BikeBinary, WalkBinary, FloorsClimbed, ElevationGain, ActiveBurnedCalories and Steps. ActivityType contains information on a detected activity in a given time period (ie, resting and walking). The remaining features indicate if, for a given time period, a person was generally physically active, running, biking, walking, how many floors or metres in altitude a person made, how many calories a person burned and the number of steps. Heart rate is collected passively using photoplethysmography (PPG). When no physical activity is detected, the Scanwatch collects heart rate data every 10 min for 30 s and provides the average heart rate for that sampling interval. As soon as physical activity is detected, the Scanwatch collects PPG-based heart rate continuously until the activity stops.

The Withings Scanwatch also allows you to perform 30-s resting-state ECGs (300 Hz) with medical-device quality. During active assessment phases, patients are asked to conduct an ECG on their Scanwatch two times a day. The resting-state ECG data are available in raw format. Withings also calculates Root Mean Square of Successive Differences (RMSSD) for resting-state ECGs as an indicator of HRV.

The TIKI app collects GPS data directly from the mobile phone’s sensor. To increase patient’ privacy and data security, GPS data are anonymised locally before saving them on the study servers by rotating the Latitude–Longitude tuples by a random angle. Like this, only mobility patterns (ie, distance travelled) but not exact locations can be inferred.

### Sample size calculation

As described in the main study protocol,^1^ the required sample size for the PREACT study was estimated (a) using simulation studies with fivefold cross-validation to yield a prediction accuracy of 75–80% and (b) using effect size calculation for group-based linear multiple regression analyses, resulting in a required sample of 585 patients. Of those, it was expected that around 80% would take part in PREACT-digital, resulting in an estimated sample size of 468 patients for T0. Based on previous studies investigating the feasibility of digital phenotyping in clinical samples,^28^ it was further expected that 75% of the sample would decide for the long version of the study, resulting in 350 patients providing data at T20 and TPost.

### Outcomes

We use different machine learning techniques to predict treatment response based on DP data. To evaluate treatment response, we implement two different criteria. First, we follow the definition of the PREACT research group to achieve comparability with other subprojects. It follows the concept of clinically significant change as introduced by Jacobson & Truax.^29^ The shift from a dysfunctional to a functional mental condition is operationalised by achieving a Brief Symptom Inventory-General Severity Index (BSI–GSI) score of less than 0.56 at T20/posttreatment assessment. Additionally, the change observed from baseline to T20/posttreatment must be reliable, as defined by the Reliable Change Index (RCI). The BSI–GSI is a self-report measure assessed cross-sectionally at T20 and TPost as part of PREACT.

Second, we will use aggregates of EMA assessments on positive and negative affect (PANAS-X; 21) collected during measurement bursts at T20 and TPost as additional indicators of treatment response as EMA-based measures were shown to capture different aspects of symptom load (28).

To account for the complexity of ID, PREACT further included disorder-specific measures as secondary outcomes: The Hamilton Anxiety Rating Scale (HAM-A; 29), the Montgomery–Åsberg Depression Rating Scale (MADRS; 30), the Yale-Brown Obsessive Compulsive Scale (Y-BOCS; 31, 32) and the Clinician-Administered PTSD Scale for DSM-5 (CAPS-5; 33, 34).

Finally, for exploratory analyses, outcomes will depend on the respective research question, that is, single-beep scores of EMA constructs or cross-sectional self-reports of ER capacities.

### Data preprocessing and feature engineering

In its raw format, passive sensing data are usually not interpretable. Thus, depending on the data source, data format and research question, different data preprocessing and aggregation steps will be necessary.

1. GPS data are collected as tuples containing coordinates (ie, Latitude and Longitude values). To clean and preprocess the data and build ML features, we follow guidelines by Müller *et al* (35). We adapt them to our event-based sampling format, where GPS was assessed when a location change was detected. From GPS data, we will calculate movement patterns in predefined timescales, that is, location entropy, distance travelled or time spent at home.
2. Passive data derived from the Withings Scanwatch will be further preprocessed where possible and necessary, as Withings does not provide raw data for most data sources (see table 1). Here, we align our preprocessing steps with guidelines for preprocessing sensor data (36). From continuous PPG-based heart ate data, we are able to build features like average HR, min/max HR and HR-zones. From the resting-state ECG,we can create more fine-grained features like HRV. For sleep, we will calculate features describing sleep quality, that is, sleep duration, proportion of sleep phases and sleep efficiency (i.e. sleep duration/ time in bed) per night. Activity features describe the amount of, or time in active or inactive states, that is, total number of steps or time spent walking/running/cycling and the amount of calories burned.
3. For EMA data, we will calculate the RMSSD (that is, for emotional inertia or ER variability), individual SD and mean values. Further feature engineering steps for EMA are described in the *Analysis* section.

Depending on the research question and granularity of data aggregation, a certain compliance rate has to be met to be included in the analysis (ie, at least 50% of completed EMA beeps). Missing data imputation will also depend on the type of data and applied analyses.

### Analysis

The analysis procedure and code will be provided on OSF and on Github (https://github.com/leona-ha/preact_digital). Data preparation, exploration and modelling will mainly be done using Python, R in the Jupyter Lab environment, conducted on the computing facility of Charité – Universitätsmedizin Berlin.

#### Data freeze, holdout and model training

To allow for exploratory analyses without risking data leakage for predictive modelling, all data collected up to November 2024 were frozen. PREACT then drew an a priori, participant-level hold-out sample comprising 20 % of the cohort, stratified by age, sex, primary diagnosis and data modality (EEG, MRI, digital-phenotyping short vs long). The remaining 80 % constitute the development data set.

Participants enrolled after November 2024 (ie, after the data freeze) are assigned to development or hold-out according to the same 80 : 20 stratified rule so that these proportions are preserved when recruitment closes in June 2025.

#### Model development

Within the development set, we apply nested, repeated, stratified fivefold cross-validation. In each outer fold, 80 % of development data form the training subset and 20 % the validation subset for hyperparameter tuning and model selection. Performance metrics are averaged across folds and repetitions to yield optimism-corrected estimates. The optimised pipeline is then evaluated on the untouched 20 % hold-out test set.

Exploratory analyses that do not involve model building are conducted exclusively within the development set to safeguard the integrity of the hold-out sample.

#### Predictive analyses

We will implement both basic and advanced models and compare subsets of data and different timeframes to balance interpretability and predictive performance. With a sample size of at least 350 participants, we have sufficient data to explore a range of methods.

There are various ways to aggregate and preprocess EMA data to include them as predictors of non-response, ranging from simple aggregates to advanced modelling techniques. We will implement dynamic structural equation models (DSEM), an advanced extension of multilevel modelling that integrates time-series dynamics with latent variable modelling.^30^ Multilevel models are considered the gold standard for longitudinal data analysis in psychology,^31^ and DSEM builds on this foundation to allow unequally spaced observations and modelling of time-lagged dependencies, making it well suited for EMA data. The Bayesian estimation approach allows for missing data under the missing at random assumption (eg, when participants skip measurements). Beyond that, DSEM models are extendable to model change in dynamic parameters across measurement bursts. Further, passive data can be flexibly integrated into DSEM to analyse cross-lagged relationships between EMA (ie, affect) and passive sensing features (ie, sleep and physical activity). The model-based framework allows us to derive dynamic parameters (eg, individual autocorrelations and cross-lagged relationships) that we will use as features for the predictive analyses.

For the prediction of NR, basic algorithms include logistic regression, support vector machines, random forests and gradient boosting, depending on the research question and predictors used. Tree-based approaches are known for their effectiveness on tabular data.^32^ Regression-based approaches are highly interpretable. All of the selected require fewer computational resources compared with deep learning models and were frequently applied in psychotherapy research, increasing comparability.^33^,^35^

Advanced modelling approaches will be selected to incorporate the hierarchical, sequential and multimodal structure of our data. These include deep learning architectures like LSTM, Transformers or ensemble learning approaches. To explore how the predictive capacities depend on the time lag, we compare the accuracies of models using subsets of available data (eg, first measurement burst vs second measurement burst) and outcome timepoints (ie, T20 vs TPost).

As outlined above, we are interested in the incremental value of DP and EMA data to less burdensome, cross-sectional self-reports. As a first step, we will construct a baseline model that relies exclusively on the cross-sectional information collected at enrolment (T0).

Predictors will mirror the variables listed in the main protocol^1^—emographics, clinical routine variables (ie, primary diagnosis, comorbidity, medication and previous CBT) and standard intake questionnaires. Separate models will be trained for our two prespecified outcomes (T20, TPost). The baseline cross-sectional models will be compared against four progressively richer model sets: (a) EMA-only models, (b) passive-sensor-only models, (c) EMA+passive-sensor models and (d) all data sources combined (T0+EMA + passive). Paired differences in area under the curve (with 95% CIs, DeLong test) will quantify improvements in discrimination of (non)-response.

To assess feature importance and increase interpretability of our models, we will implement model-agnostic SHapley values (SHAP, SHapley Additive exPlanations).

#### Exploratory analyses

Our exploratory analyses encompass statistical and inferential investigations, where models are built to explore selected mechanisms and relationships between different variables, data domains and assessment times. We further aim to validate our EMA-based TA scale using Multilevel Confirmatory Factor Analysis (MCFA). To explore trajectories of change during therapy, we will make use of clustering techniques (ie, k-means clustering) or latent class growth analysis (LCGA). Derived clusters or classes may serve as additional features for the prediction of treatment NR.

### Patient and public involvement

During the first week of study participation, we conduct a standardised interview with participants where we assess the feasibility and acceptability of our ambulatory assessments. Here, patients are also invited to suggest improvements. In addition, as part of subproject 5,^1^ patients report, among others, the burden and time required for participating in our subproject. All results regarding feasibility and acceptability will be considered when working on the follow-up proposal to this project. To elaborate strategies for dissemination of study results to participants and wider patient communities, we will conduct a congress open to the public in 2025.

## Ethics and dissemination

The study has been approved by the Institutional Ethics Committee of the Department of Psychology at Humboldt-Universität zu Berlin (approval no. 2021–01) and the Ethics Committee of Charité – Universitätsmedizin Berlin (approval no. EA1/186/22). All participants provide written informed consent after being fully informed about the study’s aims, procedures, potential risks and benefits, and they may withdraw at any time without consequence. To meet the requirements of the EU General Data Protection Regulation (“Datenschutz-Grundverordnung,” DSGVO), we have implemented a data-protection concept for prospective clinical studies and a Joint Controller Agreement covering all participating institutions, both approved by their respective data-protection officers. Participant data are pseudonymised and stored on encrypted servers with role-based access rights, audit trails, standard operating procedures and staff training; no identifiable information is shared with third parties. Data integrity, participant safety and adverse events are continuously monitored. One year after the last patient out, the dataset will be fully anonymised to support open-science practices while safeguarding privacy. De-identified data and analysis scripts will be available to researchers within the RU via a secure server and may be shared with external investigators on reasonable request under GDPR-compliant agreements and in line with participant consent. Results will be disseminated through peer-reviewed journals, presentations at national and international conferences, and lay summaries posted on institutional websites or presented at public events.

## Discussion

Within the research endeavour of the PREACT study to find predictors of non-response to CBT for ID, PREACT-digital implements therapy accompany digital phenotyping. DP complements the other subprojects by contributing dense, behavioural and physiological information from the patients everyday life, providing an ecologically valid and innovative add-on to more traditional sources of data. By integrating EMA with wearable-based passive sensing, we capture detailed, real-time information that goes beyond traditional cross-sectional snapshots. Our predictive analyses aim to determine whether advanced but computationally demanding modelling approaches improve accuracy over simpler methods, while exploratory analyses focus on uncovering potential mechanisms and subgroups that may inform tailored interventions.

A key strength of our study lies in its ecological validity: participants provide data in naturalistic settings, resulting in rich, high-frequency observations. We further benefit from a relatively large sample size and the opportunity to capture change processes over multiple time points. The integration of our project into the Research Unit allows us to compare our digital phenotypes with other data modalities and acquire a rich, comprehensive impression of participating patients. Although a few studies have integrated DP into outpatient therapy settings, this combination of different data modalities is unique to our study.

Despite these strengths, there are also key limitations that demand consideration. First, the study includes no healthy control group. As a result, we cannot directly distinguish digital patterns that are specific to clinical presentations from those also present in non-clinical populations. Future work will need case–control or population-based designs to clarify the specificity of any digital signatures identified here. Second, the extensive assessment schedule may place a burden on participants. Although we tried to keep the length of our active assessments reasonable (ie, around 3–5 min to complete it), answering eight daily questionnaires across 14 days is non-negligible. Also, participants are asked to wear their smartwatch as continuously as possible, also during nighttime. This combination of frequent questionnaires and continuous sensing may reduce adherence over time and, in the worst case, could lead some participants to withdraw from the study.

We hope to identify both robust predictors of therapy response and clinically meaningful insights that can guide personalised treatment adaptations. As the field of DP in mental health is in its infancy, our study will contribute relevant evidence regarding its feasibility in psychotherapy contexts and its informational content regarding treatment response.

Ultimately, this research has the potential to inform the development of more dynamic, precise, and patient-centred therapy approaches for ID. Early identification of individuals at risk of non-response to CBT could enable not only improved treatment allocation but also the implementation of adaptive interventions. For example, predicted non-responders may benefit from intensified care (eg, stepped care), evidence-based augmentation strategies (eg, pharmacological or mindfulness-based enhancements). DP data, in particular, may also allow the identification of symptom worsening in real time and thus trigger a suitable intervention on the smartphone (just-in-time adaptive interventions) or inform the respective therapist (blended treatment) about symptom triggers and contexts. Thus, predictive models in general, and predictive DP models in particular, could serve as a foundation for tailoring treatment pathways in a more responsive and individualised manner.
