# Supplementary material for: PREACT-digital: study protocol for a longitudinal, observational multicentre study on digital phenotypes of non-response to cognitive behavioural therapy for internalising disorders
Source: BMJ Open. 2025 Jul 10;15(7):e102392. doi: 10.1136/bmjopen-2025-102392 (PMC12258351; doi:10.1136/bmjopen-2025-102392)
Supplement: Supplementary file 1 [file bmjopen-2025-102392.reviewer_comments.pdf]

PEER REVIEW HISTORY

BMJ Open publishes all reviews undertaken for accepted manuscripts. Reviewers are asked to complete a checklist review form and are provided with free text boxes to elaborate on their assessment. These free text comments are reproduced below.

ARTICLE DETAILS

Title (Provisional)

PREACT-digital: study protocol for a longitudinal, observational multi-center study on digital phenotypes of non-response to cognitive behavioral therapy for internalizing disorders

Authors

Hammelrath, Leona; Brose, Annette; Heinrich, Manuel; Zagorscak, Pavle; Burchert, Sebastian; Langhammer, Till; Knaevelsrud, Christine

---

VERSION 1 - REVIEW

Reviewer 1

Name Joyce, Dan

Affiliation University of Liverpool Faculty of Health and Life Sciences, Primary care and Mental Health

Date 14-Apr-2025

COI None

This is an interesting approach to studying response (specifically, why people do not respond to CBT) using granular measurement (EMA and passive sensing) of clinical state. The protocol describes the sub-study (PREACT) and gives some detail of the design of the sub-study and the proposed analyses / algorithms that will be tested.

Primary outcome is defined as response (non-response) at 20 therapy sessions and at termination of therapy.

Aim to predict treatment response at T20 (after 20 sessions) and TPost (end of therapy) from EMA and passive sensing data at T0 = a 14 day assessment phase.

Hypothesis that T0 data (passive and EMA) will collectively be better at predicting response at T20 and TPost than passive or EMA alone.

Secondary hypotheses : symptom changes during CBT to be modeled as a function of EMA and passive sensing - and how they "relate" to cross sectional self-report / physiological "information"

Specific Comments:

1. On pp 15 : HRV estimated from the two 30 second ECG samples taken daily. Could the authors explain how these brief ECG samples will give a useful measure of HRV when they are essentially collected at two daily time points -- what do the authors expect this will predict within / between days ? For example, if there are two daily HRV measures, then how will a time-series of twice-daily HRV measure be analysed ?

2. pp 15. (Analysis) can the authors specify if the hold-out (validation/testing) sample will be "per participant" ? i.e. all data from those 20% of participants will be held-out wholesale (e.g. T0, T20 and TPost) ? If so, this equates to ~ 94 (for N = 468) or allowing for expected attrition, 70 (N = 350) patients. Will the hold-out samples be representative (in terms of which internalising disorder they are being treated for) ? And will other steps be taken to ensure that the distribution of the hold-out set is comparable to the training set ? As this is a multi-site trial, will the hold-out set be clustered by centre ?

3. I could not find a citation to a relevant source on DSEM which are proposed as the "gold standard" (pp 16) and "Bayesian" but it's somewhat difficult to evaluate this claim without relevant citations to the literature.

4. I would argue (pp 16) that log regression, SVMs , RFs and gradient boosting are different : logistic regression models the probability of an outcome (e.g. not directly behaving as a classifier), SVMs, RFs and gradient boosted trees are (usually) classifiers. Could the authors justify (in more detail) the choice of these methods as "classical" ML techniques to be applied ? Similarly, how are gradient boosting (ensemble of weak-learner trees) intepretable ? Some precision on this would help understand why these methods have been chosen.

5. pp 17, lines 425--430 : "apply a consistent validation strategy" -- could the authors completely specify the sample splitting proportions ? Earlier, they refer to a training and hold out set, but then describe "train, test and validations sets". If this is an internal validation approach (i.e. no new data is prospectively collected to validate the trained algorithms) then see point 2. above -- the proportion of training, validation and test data need to be more clearly specified.

6. The authors could helpfully add some detail about how a 'basic' model predicting response (at T20 and TPost) might be used as the default comparator : for example, cross-sectional (rather than EMA / passive data) collected at enrolment might be an adequate and non-burdensome (compared to a 14 day EMA / passive data) predictor of treatment response -- this might be unlikely (and I assume the motivation for exploring digital phenotyping for CBT treatment response is that there appear to be no robust predictors from cross-sectional pre-treatment data) but it would be a helpful base model to compare ESM, passive and combined ESM & passive data for gains in predictive ability.

Minor Comments

1. In the abstract, it would help to expand abbreviations such as LSTM, DSEM

2. Define "scanwatches" in the Strengths and Limitations section

3. On pp. 6 : define "T20 and TPost" at first mention.

4. On pp. 12 : define "Rmssd" on first use

**Reviewer**	**2**
**Name**	**Mason, Ava**
**Affiliation**	**University College London, Division of Psychiatry**
**Date**	**22-Apr-2025**
**COI**	**None**

This paper can provide important novel implications on predicting non-response to CBT for internalising disorders. The paper provides sufficient detail on the methods and analysis that will be conducted. Within the study procedures, the measures were clearly explained and relevant tables and figures were provided to assist with the information given. Relevant detail was provided for the analysis and dissemination of the results. Find below some minor comments to provide more clarity and emphasise the clinical implications of the study.

In the abstract (methods and analysis section), please describe what types of disorders count as 'internalising disorders'.

In the introduction (line 102), please provide examples of other forms of psychotherapy that have not found a significant difference in outcomes vs CBT. This point also raises a question as to what might be effective in those that may not be responsive to CBT, please provide information as to what these individuals may benefit from.

In the introduction (line 133)- please provide some examples of what behavioural patterns were associated with internalising disorders.

In the methods section (line 191), please provide some examples of what medical conditions would contraindicate CBT.

In the study procedures section, would we expect different predictors to affect non-response in the different internalising disorders? Eg. OCD vs PTSD. More detail should be provided on this at some point, as I have never seen all these disorders grouped into 'internalising disorders' before. More detail should also be provided on the rationale of grouping together the disorders in this way.

Within the discussion, it is important to consider the clinical implications of the study. For example, what could be provided for individuals who may not respond to CBT? It is unclear at the moment what these individuals may benefit from, and therefore what would be helpful in predicting non-response (other than delivering CBT to those that may be more likely to respond).

VERSION 1 - AUTHOR RESPONSE

Reviewer 1:

1. On pp 15 : HRV estimated from the two 30 second ECG samples taken daily. Could the authors explain how these brief ECG samples will give a useful measure of HRV when they are essentially collected at two daily time points -- what do the authors expect this will predict within / between days ? For example, if there are two daily HRV measures, then how will a time-series of twice-daily HRV measure be analysed ?	You raise a valid point! The manual ECG recordings provide us with finegrained, raw data that allow us to calculate HRV. They are an addition to the PPG-based heart rate that is collected continuously and allow the modelling of long-term trajectories. From the manual ECG recordings we will create daily aggregates, but might also include them in mixed-effect models (similarly to EMA measurements) to model within-person fluctuations in HRV over time.
2. pp 15. (Analysis) can the authors specify if the hold-out (validation/testing) sample will be "per participant" ? i.e. all data from those 20% of participants will be held-out wholesale (e.g. T0, T20 and TPost) ? If so, this equates to ~ 94 (for N = 468) or allowing for expected attrition, 70 (N = 350) patients. Will the hold-out samples be representative (in terms of which internalising disorder they are being treated for) ? And will other steps be taken to ensure that the distribution of the hold-out set is comparable to the training set ? As this is a multi-site trial, will the hold-out set be clustered by centre ?	Excuse the lack of clarity! The holdout set was defined on the superior level of the research group. We aimed for a representative split, considering age, sex, diagnosis and whether they are in the short vs. long version oft he study. The holdout set was not clustered by center, as most data was collected off-site (i.e. not in the therapy ambulances). A more detailed description was added to the manuscript
3. I could not find a citation to a relevant source on DSEM which are proposed as the "gold standard" (pp 16) and "Bayesian" but it's somewhat difficult to evaluate this claim without relevant citations to the literature.	You're correct! I adjusted the paragraph accordingly.
4. I would argue (pp 16) that log regression, SVMs , RFs and gradient boosting are different : logistic regression models the probability of an outcome (e.g. not directly behaving as a classifier), SVMs, RFs and gradient boosted trees are (usually) classifiers. Could the authors justify (in more detail) the choice of these methods as "classical" ML techniques to be applied ? Similarly, how are gradient boosting (ensemble of weak-learner trees) intepretable ? Some precision on this would help understand why these methods have been chosen.	I very much understand your point about our choice of algorithms. The alghorithms were chosen as they are frequently applied in mental health settings and thus increase comparability. Additional algorithms will be applied, depending on research question and state-of-the-art algorithms. We added more detail on why these methods were chosen. I also agree on interpretability of gradient boosting and adjusted that point.
5. pp 17, lines 425--430 : "apply a consistent validation strategy" -- could the authors completely specify the sample splitting proportions ? Earlier, they refer to a training and hold out set, but then describe "train, test and validations sets". If this is an internal validation approach (i.e. no new data is prospectively collected to validate the trained algorithms) then see point 2. above -- the proportion of training, validation and test data need to be more clearly specified.	Excuse the confusion! They very likely come, as you mentioned, from the difference between internal and external validation (as part of the research group). For internal, explorative analyses we will implement separate train-test splits within the users that are not part oft he external holdout.<br><br>The description was clarified in the method section.

6. The authors could helpfully add some detail about how a 'basic' model predicting response (at T20 and TPost) might be used as the default comparator : for example, cross-sectional (rather than EMA / passive data) collected at enrolment might be an adequate and non-burdensome (compared to a 14 day EMA / passive data) predictor of treatment response -- this might be unlikely (and I assume the motivation for exploring digital phenotyping for CBT treatment response is that there appear to be no robust predictors from cross-sectional pre-treatment data) but it would be a helpful base model to compare ESM, passive and combined ESM & passive data for gains in predictive ability.	Thanks for the comment! Yess, we will definitely compare our models using digital phenotyping data to a base models, using cross-sectional self-reports collected pre-treatment. We added this detail to the manuscript.
1. In the abstract, it would help to expand abbreviations such as LSTM, DSEM	Was adjusted.
2. Define "scanwatches" in the Strengths and Limitations section	Was adjusted.
3. On pp. 6 : define "T20 and TPost" at first mention.	Was adjusted.
4. On pp. 12 : define "Rmssd" on first use	Was adjusted.

Reviewer 2:

Comment	Response
In the abstract (methods and analysis section), please describe what types of disorders count as 'internalising disorders'.	Descrition was added
In the introduction (line 102), please provide examples of other forms of psychotherapy that have not found a significant difference in outcomes vs CBT. This point also raises a question as to what might be effective in those that may not be responsive to CBT, please provide information as to what these individuals may benefit from.	Old source was replaced with a more appropriate one; more explanations added; Alternative/Better treatment ideas: I think that is what we are trying to find out in the next step, where we compare different/new adaptations of CBT, like i.e. breathwork or ecologically mmentary interventions (i.e. small interventions on the patients smartphone in their everyday life).

In the introduction (line 133)- please provide some examples of what behavioural patterns were associated with internalising disorders.	Examples of behavioral patterns and respective studies were added

In the methods section (line 191), please provide some examples of what medical conditions would contraindicate CBT.	In the German health-care system every course of outpatient psychotherapy—including cognitive-behavioural therapy—must be preceded by a physician-completed *Konsiliarbericht* (medical consultation report). This standardized report confirms that (i) the patient has undergone a somatic evaluation, and (ii) no medical condition is present that would render psychotherapy unsafe or inappropriate. Because this mechanism is mandated nationwide, we did not pre-define an exhaustive list of contraindications in the original manuscript. Nevertheless we now included an example in our method section.
In the study procedures section, would we expect different predictors to affect non- response in the different internalising disorders? Eg. OCD vs PTSD. More detail should be provided on this at some point, as I have never seen all these disorders grouped into 'internalising disorders' before. More detail should also be provided on the rationale of grouping together the disorders in this way.	The whole research group follows a transdiagnostic approach. This is why we decided not to focus on separate diagnoses like previous studies did, but rather groups of diagnoses with shared symptom paterns, Modern dimensional models like the **HiTOP (Hierarchical Taxonomy of Psychopathology)** propose that internalizing and externalizing are two major spectra of mental illness that together account for a large portion of disorders. Internalizing disorders are characterized by inner distress – emotions or behaviors that primarily affect the individual *themselves.* We adjusted the introduction and added a source for clarification.<br><br>Definitely, we would expect predictors to differ not only for different diagnoses (i.e. like OCD or PTSD) but even within diagnostic categories, as DP data are as heterogeneous as their underyling symptoms. This is however less a question of study procedures but rather of analysis, i.e. subgroup analyses and personalized machine learning. We added a section to the analysis section, explaining how we'll deal with these heterogenities.
Within the discussion, it is important to consider the clinical implications of the study. For example, what could be provided for individuals who may not respond to CBT? It is unclear at the moment what these individuals may benefit from, and therefore what would be helpful in predicting non-response (other than delivering CBT to those that may be more likely to respond).	Thank you for the important comment, We added a section at then end of the discussion explaining pontential clinical use cases.

VERSION 2 - REVIEW

Reviewer 1

Name Joyce, Dan

Affiliation University of Liverpool Faculty of Health and Life Sciences, Primary care and Mental Health

Date 16-Jun-2025

COI

Outstanding queries from first review completed.
